# Binge Eating Disorder (BED) in Relation to Addictive Behaviors and Personality Risk Factors

**DOI:** 10.3389/fpsyg.2017.00579

**Published:** 2017-04-25

**Authors:** Caroline Davis, Laura Mackew, Robert D. Levitan, Allan S. Kaplan, Jacqueline C. Carter, James L. Kennedy

**Affiliations:** ^1^Kinesiology & Health Science, York University, TorontoON, Canada; ^2^Department of Psychiatry, Centre for Addiction and Mental Health, University of Toronto, Toronto, ONCanada; ^3^Department of Psychology, Memorial University of Newfoundland, St. John’sNF, Canada

**Keywords:** binge eating disorder, personality risk, addictive behaviors

## Abstract

While there is good evidence that binge eating disorder (BED) is linked to higher-than-expected use of a broad range of addictive behaviors, mechanisms underlying this association are not well understood. Using a mediation-analytical approach with three age- and gender-matched groups – overweight/obese adults with (*n* = 42) and without (*n* = 104) BED, and normal-weight control participants (*n* = 73) – we tested the hypothesis that adults with BED would engage in more addictive behaviors and have higher scores on a personality-risk index than the two control groups. We also anticipated that the relationship between BED and addictive behaviors would be mediated by a high-risk personality profile. The predicted mediation effect was strongly supported. Contrary to expectation, BED participants did not engage in more addictive behaviors or have higher personality-risk scores than their weight-matched counterparts. However, both overweight/obese groups did have significantly higher scores than the normal-weight group. The relationships among personality risk, elevated body mass index (BMI), and addictive behaviors have important clinical implications, especially for treatments that target psycho-behavioral intervention for compulsive overeating and substance-use disorders.

## Introduction

Compulsive overeating, or what we call binge eating disorder (BED) in some cases, has many clinical parallels with conventional substance-use disorders, including an increasing feeling of ‘loss of control’ even in the face of adverse consequences (Davis and Carter, 2009). Typically, individuals also experience an overwhelming desire for certain (mostly highly palatable) foods, which often triggers relapses when one endeavors to restrain from binging behaviors – evidence which prompted the inclusion of “cravings” in the diagnostic criteria for BED in the most recent version of the *Diagnostic and Statistical Manual of Mental Disorders* (American Psychiatric Association, 2013). A recent body of research also demonstrates psychobiological similarities between the two conditions (Banca et al., 2016; Reichelt et al., 2016). For instance, both conditions implicate neuropathophysiological systems that regulate reward sensitivity, attentional biases, impaired cognitive function, and executive-function deficits (Guido, 2015; Kessler et al., 2016).

In particular, BED has been linked to high reward responsiveness as indicated by evidence (i) that ventral striatal activity during the anticipation of a reward was inversely related to binge-eating abstinence after treatment (Balodis et al., 2014), (ii) that those with BED had a higher genetic profile reflective of stronger brain-dopamine signaling strength compared to weight-matched controls (Davis et al., 2012), and (iii) that eye-gazing duration for food images was greater in adolescents with BED than in a matched-control group (Schmidt et al., 2016). Similar neurobiological findings have been seen in those who abuse a broad range of substances (Filbey et al., 2012) where high novelty-seeking is a key psycho-behavioral trait, which in turn is linked to various reward-related neurobiological characteristics (Silveri et al., 2016). While cross-sectional brain-imaging data may indeed identify various neurotoxic effects of heavy alcohol and drug use, it is also clear that “neurobiological signatures” (Silveri et al., 2016) can be precipitating factors for drug use, as indicated by prospective evidence that high reward sensitivity predicted earlier onset of substance use in adolescents – albeit only in those with low inhibitory control (Kim-Spoon et al., 2016).

It has been argued that in some cases, BED may itself be an addiction disorder whereby severe cases with respect to compulsiveness and frequency may reflect an abuse of, and dependence on, hyper-palatable substances. Such views are based, to some extent, on evidence that processed foods high in sugar, fat, and salt have brain-responsive properties very similar to those of other addictive drugs (Gearhardt et al., 2011). There is also substantial overlap between BED and the so-called ‘food addiction’ construct (Davis, 2016a). In addition, there is some evidence that BED and conventional addiction disorders co-occur with a greater frequency than would be found for addiction disorders in the general population. For instance, binge eaters were more likely to use all types of addictive substances compared to controls (Ross and Ivis, 1999; Ferriter and Ray, 2011) and this relationship did not seem to be moderated by sex (Ross and Ivis, 1999). A recent large-sample study of adult men and women with BED also found a 27% life-time prevalence of alcohol and substance-use disorder (Becker and Grilo, 2015). Other evidence suggests that co-morbid BED and substance-use disorders are associated with a more severe form of BED (Peterson et al., 2005).

In spite of these findings, it is important to acknowledge an alternative hypothesis which posits that overeating may compete with addictive substances for brain reward sites, and thereby result in lower prevalence rates of drug use and abuse (Kleiner et al., 2004). While there is some support for this viewpoint, the evidence is based largely on alcohol-related research. For example, a few studies have reported that body mass index (BMI) and alcohol consumption are inversely correlated based on self-report data for both variables (Kleiner et al., 2004; Gearhardt and Corbin, 2009). However, a potential confound with these studies is the fact that, unlike most addictive substances such as cocaine or nicotine, alcohol contains calories – a factor which may contribute to the negative relationship with food consumption.

### Mechanisms Linking BED and Substance-Use Disorders

Although links between BED symptomatology and substance use/abuse are well-documented, as described above, there has been little information about mechanisms that might foster this connection. One strategy is to examine the role of stable personality traits. This may be a promising approach since BED and addiction disorders have many psychological correlates and risk factors in common. For instance, both are associated with greater than expected levels of anxiety and depression (Latvala et al., 2009; Brownley et al., 2016; Jung et al., 2016; Keski-Rahkonen and Mustelin, 2016) and with personality traits such as impulsiveness and sensation seeking (Conrod et al., 2013; Lee-Winn et al., 2016). Accumulating evidence has also identified poorer interpersonal skills, avoidance of emotional expression, and a diminished ability to cope with negative feelings in those with clinically significant overeating compared to their control counterparts (Berger et al., 2014; Ivanova et al., 2015). In addition, significant associations have also been established between attachment avoidance and attachment insecurity, and binge eating (Tasca and Balfour, 2014; Shakory et al., 2015). It is possible therefore that the use of addictive behaviors may be a form of self-medication to ameliorate the effects of stressful social interactions and other distressful life events in those with BED (Besson and Forget, 2016).

The current study was designed to address two specific research questions. First of all, we tested the hypothesis that the use of a broad range of potentially addictive behaviors would be greater in those with BED compared to weight-matched controls who do not binge eat, and to a group of normal-weight control participants. It was also predicted that those with BED would have higher scores on a high-risk personality profile compared to their weight- and age-matched counterparts, and to the normal-weight controls. Finally, it was expected that the links between both behaviors would be mediated by a high-risk personality profile.

## Materials and Methods

### Participants

Adults between the ages of 25 and 47 years, who met the DSM-V criteria for BED (*N* = 42; females = 36), were recruited from the community via posters placed in many public buildings, as well as from newspaper advertisements, and online classified advertising services like Kijiji. A non-binging obese (BMI > 30) control group (*N* = 104; females = 75) and a normal-weight (BMI between 18.5 and 24.9) control group (*N* = 73; women = 49) were recruited in the same manner. While there were more woman than men in the study, the proportion of each in the three groups did not differ significantly (χ^2^ = 4.78; *p* = 0.092). The sample was largely Caucasian (82%).

### Measures

**Personality Risk** was modeled as a composite variable comprising total scores from three personality factors associated with impulsive responding, high reward sensitivity, and anxiousness and negative affect. The well-validated, 30-item *Barratt Impulsivity Scale* [BIS] (Patton et al., 1995), identifies aspects of the impulsivity construct such as non-planning and the tendency to act rashly and to make quick decisions. Reward Sensitivity was assessed by the *Reward Subscale* [RS] of the *Sensitivity to Punishment and Sensitivity to Reward Questionnaire* [SPSRQ] (Torrubia et al., 2001), which comprises 24 forced-choice items reflecting the respondent’s approach responses under various conditions of reward. This scale was developed to assess the behavioral activation system [BAS] of Gray’s psychobiological model of personality (Gray, 1987, 1990). Addictive Personality Traits were assessed by the 32-item *Addiction Scale* [AS] of the *Eysenck Personality Questionnaire-Revised* [EPQ-R] (Eysenck and Eysenck, 1991). This scale was derived empirically by identifying those items of the EPQ-R which differentiated male drug addicts from normal controls (Gossop and Eysenck, 1980). In addition to studies with drug addicts (Sigurdsson and Gudjonsson, 1995), this scale has been validated with groups of problem drinkers (Ogden et al., 1989), pathological gamblers (Clarke, 2003), and those with disordered eating (Davis and Claridge, 1998; Davis et al., 2008). The scale items are weighed toward impulsivity, as well as anxiousness, neuroticism, and negative mood.

The three variables described above were moderately correlated, as expected (*r* between 0.26 and 0.42; all *p*-values < 0.0001). A factor score was calculated using Principal Component Analysis [PCA] (SPSS Version 23). Total scores, not individual items, were entered into the PCA. The analysis extracted only one component with an Eigenvalue > 1. The three factor loadings ranged between 0.70 and 0.81, and the variance accounted for by the extracted component was 57%. In subsequent analyses, the derived factor scores for each participant were used to reflect the latent variable.

**Addictive Behaviors** were assessed by the *Shorter PROMIS Questionnaire* (Christo et al., 2003), a self-report instrument for the concurrent measurement of 16 addictive and/or excessive behaviors. Each subscale comprises 10 statements that the respondent endorses on a 6-point scale from 0 (“not like me”) to 5 (“like me”). The items for each scale reflect the common characteristics of addictive behaviors, such as use for effect, one’s protection of supply, a preoccupation with the substance or activity, using more than the individual intended, and one’s increased capacity or tolerance for the behavior. For the purpose of the current study, a total score was created by summing the items for the following seven subscales: caffeine, recreational drugs, sex, nicotine, prescription drugs, shopping/spending, and alcohol. Other subscales such as “compulsive helping – dominant/submissive” and “relationship – dominant/submissive” were deemed insufficiently related to conventional addiction disorders to be included in the aggregate score. Scores on the total score could range from 0 to 350, with higher scores indicating greater frequency or severity of use.

### Procedures

Participants in the three groups were initially screened during a structured telephone interview and excluded if they reported any serious medical conditions, were not fluent in English, were pregnant or had given birth within the previous 6 months, and were currently being treated for, or had a history of any psychiatric disorders (excluding a history of unipolar depression or BED). An appointment at the hospital research laboratory was then booked for eligible participants. On the assessment day, informed consent was obtained and participants were screened for Axis I diagnosis in a formal clinical interview using the Structured Clinical Interview for DSM disorders (SCID) carried out by trained personnel, and based on the criteria specified in the DSM-IV^1^. Participants then completed the questionnaire measures, after which height and weight were taken with participants wearing light indoor clothing and standing in stocking feet. At the end of the study, participants were paid a stipend for their participation. The data reported in this study are part of a larger study.

### Statistical Analyses

**FIGURE 1:**
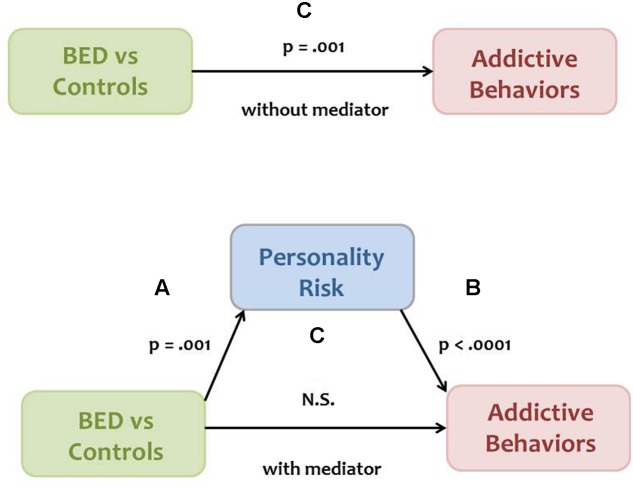
**A model proposing that a high-risk personality profile mediates the relationship between binge eating disorder (BED) status and frequency/severity of addictive behaviors**. The resulting *p*-values for paths A and C, and for paths B and C’, were derived from analysis of variance (ANOVA) and least squares regression, respectively.

The mediation model explained in the Section “Introduction” was analyzed according to the approach described by Baron and Kenny (1986). Mediation is present when the following four conditions are met. The independent variable (Group) is significantly associated with the proposed mediator (Personality Risk), depicted as ***Path A*** in **Figure 1**. Personality Risk is significantly related to the dependent variable (Addictive Behaviors), shown as ***Path B*** in **Figure 1**. The independent Group variable is significantly related to Addictive Behaviors, shown as ***Path C*** in the model. Finally, the relationship between Group and Addictive Behaviors is substantially minimized – or becomes non-significant – when the proposed mediator, Personality Risk, is added as a covariate in the ANOVA analysis depicted as ***Path C’*** in **Figure 1**. Indirect effects were assessed using PROCESS for SPSS (Hayes, 2013). Bias corrected bootstrap confidence intervals were derived at the 95% level with resamples set to 1000.

## Results

**Table 1** presents the means and standard deviations for all quantitative variables included in the analyses, in addition to age and BMI, listed separately for the BED group, the obese control (OC) group, and the normal weight control (NWC) group. The table also includes *F-* and *p*-values for one-way analysis of variance (ANOVA) comparisons among groups. Results indicated that the three groups did not differ from each other with respect to age. As expected, the groups did differ in BMI. *Post hoc* comparisons using the least significant difference (LSD) test indicated, as expected, that the NWC group had a significantly lower BMI compared to the BED and OC groups (*p* < 0.0001 in both cases), who did not differ from each other (*p* = 0.512).

**Table 1:** Means and standard deviations (SD) for all quantitative variables, listed separately for BED, NWC, and OC, and *F*- and *p*-values for one-way ANOVA comparisons among the three groups.

	BED	OC	NWC		
Variable	Mean [*SD*]	Mean [*SD*]	Mean [*SD*]	*F*	*p*
Age	32.9 [5.8]	33.2 [6.8]	31.0 [5.8]	2.6	0.075
BMI	37.9 [5.5]	37.3 [6.2]	22.5 [1.9]	216.4	<0.0001
Personality Risk (Factor Score)	0.3 [0.9]	0.1 [0.9]	-0.4 [1.0]	7.1	0.001
Addictive Behaviors	70.0 [38.0]	64.0 [39.6]	47.9 [31.7]	6.4	0.002

*BED, binge eating disorder; NWC, normal weight control; OC, obese control; ANOVA, analysis of variance*.

### Model Testing

#### Path A

**FIGURE 2:**
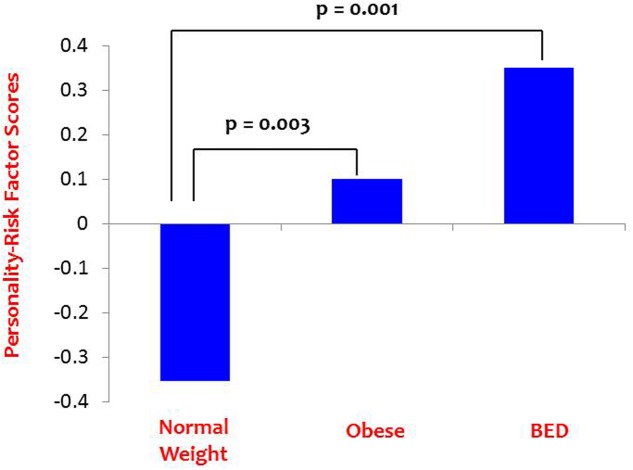
**Path A results: Personality-risk differences among the BED and control groups**.

***Path A*** was tested using a one-way ANOVA with Personality Risk as the dependent variable and Group as the independent variable, as seen in **Table 1**. The main effect was statistically significant, and LSD *post hoc* comparisons indicated that the NWC group had significantly lower Personality-Risk scores than the BED group (*p* = 0.001) and the OC group (*p* = 0.003), who did not differ from each other (*p* = 0.266). These results are depicted in **Figure 2**.

#### Path B

***Path B*** was tested by regressing Addictive Behaviors on the Personality-Risk factor score, and results indicated a significant positive association between the two variables (*r* = 0.564; *p* < 0.0001).

#### Path C

**FIGURE 3:**
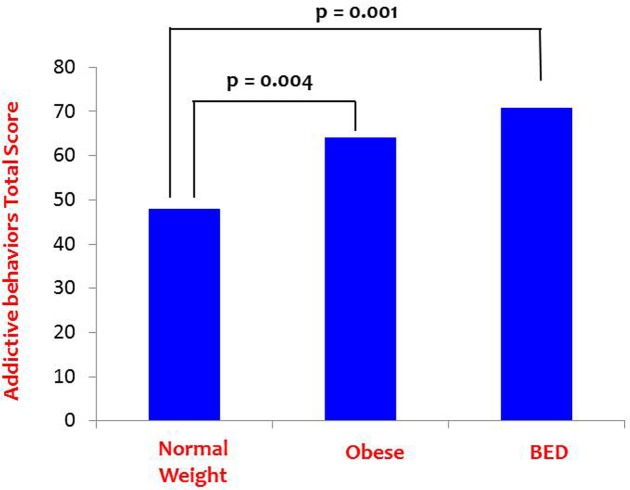
**Path C results: Addictive behavior differences among the BED and control groups**.

***Path C*** (without the mediating variable) was tested using a one-way ANOVA with Addictive Behaviors as the dependent variable and Group as the independent variable. There was a significant Group main effect as indicated in **Table 1**, and LSD *post hoc* comparisons again indicated that the NWC group reported significantly lower scores on the composite Addictive Behaviors variable compared to the BED group (*p* = 0.001) and the OC group (*p* = 0.004), who did not differ from each other (*p* = 0.315).^2^ These findings are shown graphically in **Figure 3**.

#### Path C’

In the final step, ***Path C’*** was tested by repeating the analysis described in Section “Path C,” however, this time the proposed mediator (Personality Risk) was included as a covariate in the model. Results indicated that Personality Risk was a highly significant predictor in the model, but that the Group main effect no longer contributed significantly to the variance in Addictive Behaviors. See **Table 2** for the summary statistics for this analysis.

**Table 2:** Summary statistics for the one-way ANOVA with Addictive Behaviors as the dependent variable and the Personality Risk factor score as a covariate in the model.

Source	*df*	Mean Squares	*F*	*p*-value
Intercept	1	708865.9	732.0	<0.0001
Personality Risk	1	80723.3	83.4	<0.0001
Group	2	1655.8	1.7	0.183
Error	207	968.3		
Total	211			

#### Tests of Indirect Effects between the Group Variable and Addictive Behaviors

Given the significant associations described above in paths A, B, and C, tests of indirect effects between the Group variable and Addictive Behaviors were carried out. Bootstrapping was conducted using the PROCESS Marco for SPSS with 1000 bootstrap resamples and 95% confidence intervals [CI] (Hayes, 2013). An indirect effect is considered significant when the CI does not contain zero (Preacher and Hayes, 2004). Bootstrap estimate results were statistically significant (Effect = 4.27, *SE* = 1.63) with a 95% CI from 1.04 to 7.67. These results provide evidence for a significant difference between the total effect (Path C) and the mediation pathway – that is, that the predictive power of Group on Addictive Behaviors is lessened with the inclusion of Personality Risk in the model signifying partial mediation. The percent mediation value was 52.04.

## Discussion

Contrary to our main-effect predictions, there was no significant difference between overweight/obese individuals with and without BED regarding their use of addictive behaviors, although both groups had significantly higher scores than the normal-weight control group. These findings may imply that addictive behaviors are linked to elevated BMI – and thereby to sustained overconsumption relative to energy expenditure – **irrespective** of the pattern of overeating. Such speculation has some intuitive appeal since varied patterns of consumption are also found in all substance-use disorders. For instance, while some individuals who consume alcohol to excess display bouts of regular binge-drinking followed by periods of abstinence, others consume the same amount of alcohol, but show more continual drinking throughout most days (e.g., Rehm et al., 2003). Similarly, there are various patterns of overeating that are relevant to weight gain and obesity, and each of these may have the capacity to promote addictive tendencies toward food (Davis, 2016a). Finally, our finding that BMI was *positively* associated with a broadly represented measure of addictive behaviors, does not support the view that food and other addictive behaviors compete for the same brain-reward sites (Kleiner et al., 2004).

In the current study, both overweight groups also had higher personality-risk scores than the normal-weight control group, although they too did not differ significantly from each other. Although there is some evidence that BED is associated with greater psychopathology than their weight-matched counterparts without BED (Ivezaj et al., 2016; Lo Coco et al., 2016), there were no differences between the two groups concerning the specific personality traits (viz., impulsivity, reward sensitivity, and anxiety-proneness) assessed in our study.

In support of our mediation hypothesis, however, we did find that the significant association between the independent variable (BED-status groups) and addictive behaviors was mediated by the high-risk personality profile – a constellation of traits associated with addictive behaviors in previous research (Mackinnon et al., 2014; Davis et al., 2015). In other words, and as demonstrated statistically, the association between the independent variable (group) and the dependent variable (addictive behaviors) was no longer significant when personality risk was added as a covariate in the model. We propose that the traits comprising the high-risk profile may enhance vulnerability to addictive behaviors via separate, but interconnected, mechanisms. For example, impulsiveness may be more likely to foster a proclivity for risky behaviors and a diminished capacity for appropriate restraint, while anxiousness and negative mood may increase the reinforcing potential of these behaviors as a strategy for coping with the deleterious effects of stress.

Theories of personality-risk for addiction – or what is frequently called an ‘*addictive personality*’ (see Davis, 2016b for a review) – are generally embedded in a biomedical model. As such, attempts to provide a tangible description of psychological vulnerability have relied largely on behavioral and biological constructs (Clark, 2015). Identifying the components of this alleged personality profile has proved difficult, however, due to challenges disentangling the antecedent and consequential factors in its formation. That is, it may be that the identified traits are a *result* of the addictive behaviors rather than a *predisposing* set of factors (e.g., Amodeo, 2015). For instance, it is well-established that excessive consumption of addictive substances (including hyper-palatable foods) fosters brain alterations that increase the very symptoms which define the disorder, such as compulsive use, strong cravings, and depressive mood during abstinence (Wilhelm et al., 2014; Volkow et al., 2016). On the other hand, it is also clear that addictions are substantially heritable, that risk is non-specific to particular addictive substances or behaviors, and that the common vulnerability mechanisms are shared genetically with certain personality traits (Kendler et al., 2003; Davis and Loxton, 2013; Vanyukov et al., 2015). In addition, it is important to note that impulsivity is highly pervasive in many aspects of psychopathology as highlighted by its association with 18 psychiatric diagnoses in the *Diagnostic and Statistical Manual of Mental Disorders – Fifth Edition [DSM-5]*(American Psychiatric Association, 2013; Sperry et al., 2016). In isolation – and similar to the presence of anxiety – it is therefore a fairly non-specific risk factor for a myriad of mental health disorders. And finally, we know that addiction disorders are very heterogeneous, and that any particular trait only describes a proportion of the individuals with these conditions (Szalavitz, 2015).

In addition to the strengths of this research, it is important to address its limitations in the hopes that future research can extend and replicate the current findings. Foremost was our inability to examine male–female differences and whether sex/gender moderates the relationships we identified. In particular, the sample of individuals with BED had an insufficient number of male participants to adequately power an appropriate moderation-mediation analysis. Going forward, and given the pronounced female bias in the prevalence of BED – estimates vary from a 2:1 to a 6:1 ratio (Ágh et al., 2015) – selective recruitment will be necessary to provide balanced samples according to male–female ratios. It is also important for future research in this field to place emphasis on longitudinal investigations in order to differentiate antecedent/risk factors from those which derive as a consequence of the behaviors themselves.

In summary, we found that a high-risk (‘addictive’) personality profile mediates the relationship between BED status and the use/abuse of a broad range of addictive behaviors. However, we found no evidence that overweight/obese adults with BED engaged in significantly more addictive behaviors, or had a more addictive personality, than their weight-matched counterparts. The mediational role of personality risk factors has important clinical implications, especially concerning the recent evidence from a randomized control trial, which demonstrated that a personality-targeted prevention program for adolescence was significantly more effective in reducing alcohol use and misuse than a conventional drug-education program (Conrod et al., 2013).

## Ethics Statement

The procedures reported in this study were approved by the Research Ethics Boards relevant to the institutional affiliations of the authors, and were carried out in accordance with the Declaration of Helsinki.

## Author Contributions

CD conceived of the project and oversaw the data collection. She also took the lead in drafting the manuscript. RL, AK, JC, and JK contributed to the data collection and interpretation of the data. LM contributed to the data analysis and writing of the manuscript.

## Conflict of Interest Statement

The authors declare that the research was conducted in the absence of any commercial or financial relationships that could be construed as a potential conflict of interest.

## Footnotes

1 With respect to BED diagnosis, we used a modified set of criteria for binge frequency and duration-of-occurrence (viz., at least one episode a week over a period of 6 months). This criterion is similar to what is now specified in the DSM-5).

2 As an auxiliary analysis, we assessed group differences on the seven addictive behavior scales individually. Only the shopping (*p* < 0.0001) and the nicotine (0.008) subscales reached statistical significance. The pattern of *post hoc* comparisons, using the least significant difference test, was the same for both variables. The normal-weight control group had lower scores than the BED (*p* < 0.0001 and *p* = 0.003, respectively) and the obese-control groups (*p* < 0.0001 and *p* = 0.038, respectively), who did not differ from each other (*p* = 0.129 and *p* = 0.138, respectively).
